# Tranexamic acid in bleeding trauma patients: an exploration of benefits and harms

**DOI:** 10.1186/s13063-016-1750-1

**Published:** 2017-01-31

**Authors:** Ian Roberts, Phil Edwards, David Prieto, Miland Joshi, Abda Mahmood, Katharine Ker, Haleema Shakur

**Affiliations:** 1 Clinical Trials Unit, London School of Hygiene & Tropical Medicine, Keppel Street, London, WC1E 7HT UK; 2 Catholic University of Murcia, Murcia, Spain

## Abstract

**Background:**

The CRASH-2 trial showed that tranexamic acid (TXA) administration reduces mortality in bleeding trauma patients. However, the effect appeared to depend on how soon after injury TXA treatment was started. Treatment within 3 h reduced bleeding deaths whereas treatment after 3 h increased the risk. We examine how patient characteristics vary by time to treatment and explore whether any such variations explain the time-dependent treatment effect.

**Methods:**

Exploratory analysis were carried out, including per-protocol analyses, of data from the CRASH-2 trial, a randomised placebo-controlled trial of the effect of TXA on mortality in 20,211 trauma patients with, or at risk of, significant bleeding. We examine how patient characteristics (age, type of injury, presence or absence of head injury, Glasgow coma scale (GCS), systolic blood pressure and capillary refill time) vary with time to treatment and use univariable (restriction) and multivariable methods to examine whether any such variations explain the time-dependent effect of TXA. If not explained by differences in patient characteristics, we planned to conduct separate prespecified subgroup analyses for the early benefit and late harm.

**Results:**

There was no substantial variation in age or capillary refill by time to treatment. However, the proportion of patients with blunt trauma, the proportion with head injury and mean systolic blood pressure increased as time to treatment increased. Mean GCS decreased as time to treatment increased. Analyses restricted to patients with blunt trauma, those without head injury and those with a systolic blood pressure <100 mmHg showed that these characteristics did not explain the time-dependent treatment effect. In a multivariable analysis the interaction with time to treatment remained highly significant (*p* < 0.0001). Separate subgroup analyses that examine how the benefits of early TXA treatment and the harms of late TXA treatment vary by systolic blood pressure (≤75, 76–89, >89 mmHg); GCS (severe 3–8, moderate 9–12, mild 13–15); and type of injury (penetrating versus blunt) showed no significant heterogeneity.

**Conclusions:**

The time-dependent effect of TXA in bleeding trauma patients is not explained by the type of injury, the presence or absence of head injury or systolic blood pressure. When given within 3 h of injury, TXA reduces death due to bleeding regardless of type of injury, GCS or blood pressure.

**Trial registration:**

ClinicalTrials.gov, NCT00375258. Registered on 11 September 2006.

**Electronic supplementary material:**

The online version of this article (doi:10.1186/s13063-016-1750-1) contains supplementary material, which is available to authorized users.

## Background

The CRASH-2 trial showed that administration of tranexamic acid (TXA) to bleeding trauma patients who are within 8 h of injury reduces death due to bleeding (relative risk (RR) = 0.85, 95% CI 0.76 to 0.96) and all-cause mortality (RR = 0.91, 95% CI 0.85 to 0.97) [1]. However, prespecified subgroup analyses showed that the effect of TXA depends on the time interval between the injury and the start of treatment [2]. Treatment initiated within 3 hours﻿ of injury significantly reduced death due to bleeding (RR = 0.72, 95% CI 0.63 to 0.83), whereas initiation after 3 hours increased the risk (RR = 1.44, 95% CI 1.21 to 1.84).

Although we expected that early treatment would be more effective, we did not expect late treatment to increase the risk of death due to bleeding. On biological grounds, we anticipate treatments to have broadly similar effects on a given outcome and should be wary of claims that a treatment is highly beneficial in one subgroup but harmful in another [3]. The strong interaction with time to treatment suggests that patients treated within 3 h of injury are different to those treated after 3 h in ways that are relevant to the mechanism of action of TXA [3]. For example, if TXA was harmful in patients with head injury and such patients were underrepresented in those treated within 3 h but overrepresented in those treated after 3 h, this might explain the time-dependent treatment effect. We examine how patient characteristics vary by time to treatment in the CRASH-2 trial and explore whether any such variations explain the time-dependent treatment effect.

## Methods

### Study design and patients

The CRASH-2 trial was a randomised placebo-controlled trial of the effect of TXA on death and vascular occlusive events in adult trauma patients with, or at risk of, significant bleeding within 8 h of their injury. After collecting baseline data on patient characteristics, patients were randomly allocated to TXA (loading dose 1 g over 10 min followed by an infusion of 1 g over 8 h) or matching placebo. The primary outcome was death in hospital within 4 weeks of injury. We classified cause of death into the following categories: bleeding, vascular occlusion (myocardial infarction, stroke, and pulmonary embolism), multiorgan failure, head injury and other. Follow-up data were available for 99 · 6% of patients. The trial was conducted in 274 hospitals in 40 countries. We have previously published a detailed description of the trial [1, 2].

### Analysis

We described graphically the relationship between time since injury and the following patient characteristics: mean age, proportion with blunt trauma, proportion with head injury, mean Glasgow Coma Scale (GCS) score, mean systolic blood pressure (SBP) and mean capillary refill time. If a characteristic varied by time to treatment, we examined the impact of this variable on the time to treatment interaction using univariable (restriction) and multivariable methods.

For early and late treatment, we examined separately how the effects of TXA treatment on deaths due to bleeding vary according to patient characteristics in the following subgroup analyses: SBP (≤75, 76–89, >89 mmHg); GCS score (severe 3–8, moderate 9–12, mild 13–15); type of injury (penetrating versus blunt). All of these analyses were prespecified in the trial protocol. Heterogeneity in effects across subgroups was assessed by a χ^2^ test. We prespecified that unless there was strong evidence against the null hypothesis of homogeneity of effects (i.e. *p* < 0.001), the overall relative risk would be considered the most reliable guide to the approximate relative risks in all subgroups. Precision was quantified using 95% confidence intervals (CI).

We assessed the evidence for interaction between TXA and time since injury after adjusting for any independent effects of SBP, GCS score and type of injury, as well as any interactions between TXA and these characteristics. For this we fitted multivariable logistic regression models and used likelihood ratio tests.

## Results

**Fig. 1:**
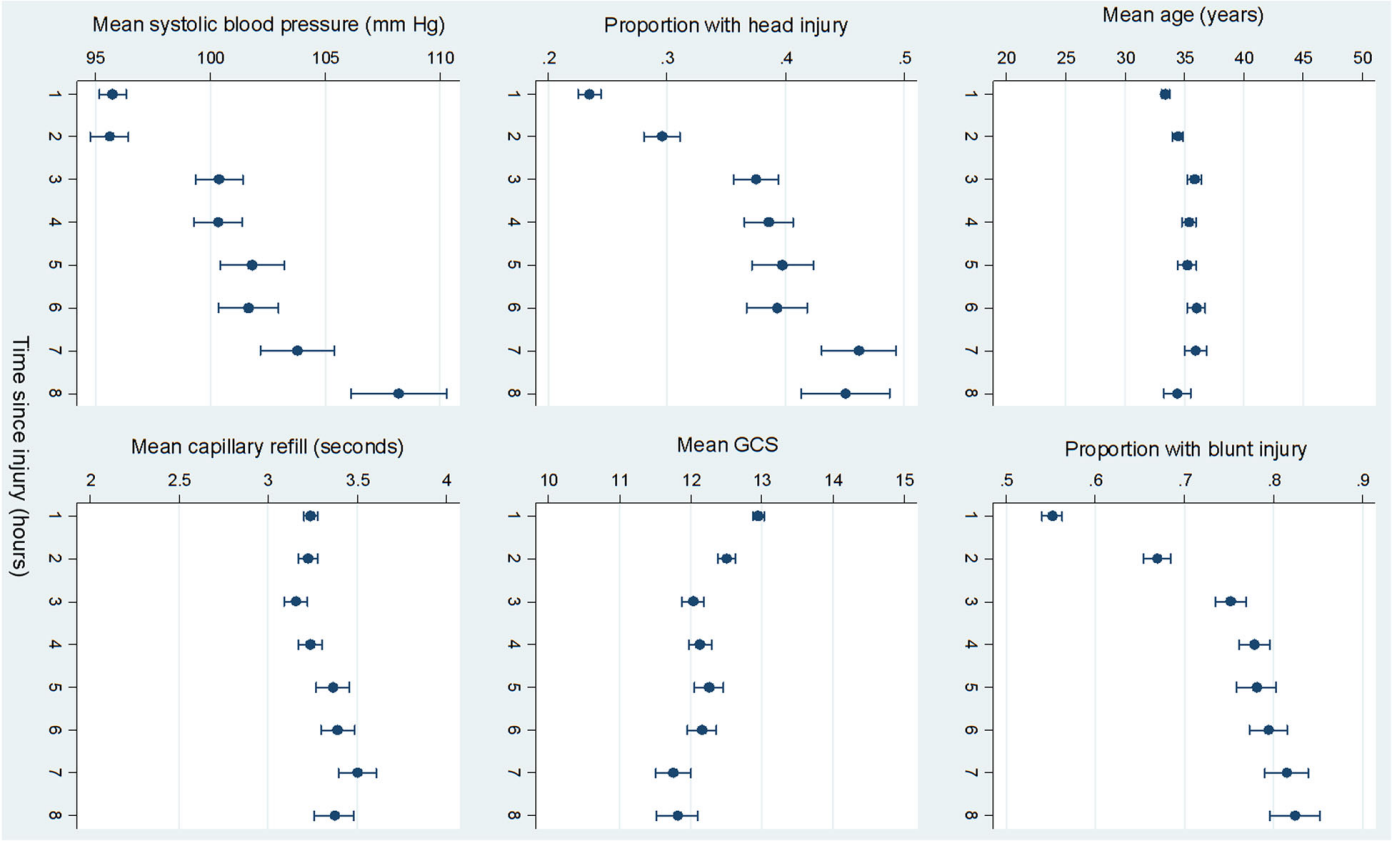
Patient characteristics by time from injury to treatment

Figure 1 shows the relationship between patient characteristics and time from injury to treatment. There was little variation in mean age or mean capillary refill by time to treatment. However, the proportion of patients with blunt trauma, the proportion with head injury and mean SBP increased as time to treatment increased. Mean GCS score decreased with increasing time to treatment.

Table 1 shows the effect of TXA on death due to bleeding stratified by time to treatment. There was strong evidence of a time to treatment interaction. To explore whether the time to treatment interaction was explained by type of injury, we restricted the analyses to patients with blunt trauma. Strong evidence of a time to treatment interaction remained (*p* = 0.0001). Among patients with blunt trauma, treatment initiated within an hour of injury reduced the risk of death due to bleeding (RR = 0.68; 95% CI 0.53 to 0.87), as did treatment initiated between 1 and 3 h (RR = 0.76; 95% CI 0.59 to 0.99), whereas treatment beyond 3 h increased the risk of death due to bleeding (RR = 1.48; 95% CI 1.12 to 1.96).

**Table 1:** Effect of tranexamic acid (TXA) on death due to bleeding by time from injury to initiation of treatment

Time to treatment (h)	TXA allocated	Placebo allocated	Risk ratio (95% CI)
≤1	198/3747 (5 · 3%)	286/3704 (7 · 7%)	0 · 68 (0 · 57–0 · 82)
>1 to ≤3	147/3037 (4 · 8%)	184/2996 (6 · 1%)	0 · 79 (0 · 64–0 · 97)
>3	144/3272 (4 · 4%)	103/3362 (3 · 1%)	1 · 44 (1 · 12–1 · 84)

Interaction χ^2^ = 23 · 516, *p* < 0 · 0000

To explore whether the time to treatment interaction was explained by the presence or absence of head injury, we restricted the analyses to patients without head injury. Strong evidence of an interaction remained (*p* = 0.0001). Among patients without head injury, treatment initiated within an hour of injury reduced the risk of death due to bleeding (RR = 0.64; 95% CI 0.52 to 0.78), as did treatment initiated between 1 and 3 h (RR = 0.71; 95% CI 0.55 to 0.91), whereas treatment beyond 3 h increased the risk of death due to bleeding (RR = 1.41; 95% CI 1.05 to 1.90).

To explore whether the time to treatment interaction was explained by SBP, we restricted the analyses to patients with a SBP <100 mmHg. Strong evidence of an interaction remained (*p* = 0.0001). Among patients with SPB <100 mmHg, treatment initiated within an hour of injury reduced the risk of death due to bleeding (RR = 0.69; 95% CI 0.58 to 0.83), as did treatment initiated between 1 and 3 h (RR = 0.84; 95% CI 0.67 to 1.04), whereas treatment beyond 3 h increased the risk of death due to bleeding (RR = 1.52; 95% CI 1.15 to 2.00).

After adjusting for any independent effects on deaths due to bleeding of SBP, GCS score and type of injury, the time to treatment interaction remained strong and highly statistically significant (*p* < 0.0001). As a sensitivity analysis, we tested for confounding of the time to treatment interaction (Appendix). After controlling for confounders, the treatment-time interaction remained in the case of models with SBP and type of injury, although the interaction was less obvious after controlling for GCS. The extent to which the treatment group by time since injury interaction varies by subgroup in illustrated pictorially in the Appendix.

**Fig. 2:**
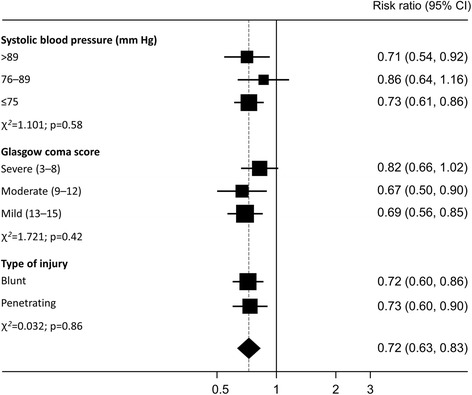
Effects of early tranexamic acid (TXA) treatment stratified by systolic blood pressure (SBP), Glasgow Coma Scale (GCS) score and type of injury

**Fig. 3:**
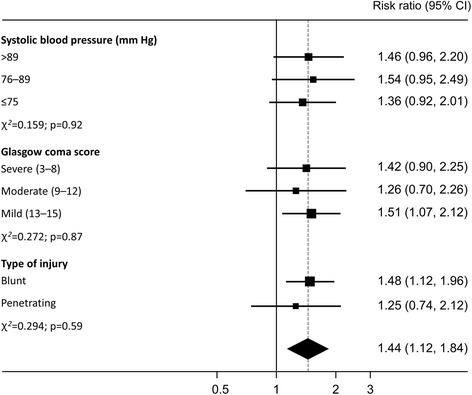
Effects of late tranexamic acid (TXA) treatment stratified by systolic blood pressure (SBP), Glasgow Coma Scale (GCS) score and type of injury

Because the time to treatment interaction could not be explained by differences in the characteristics of early and late-treated patients, we conducted subgroup analyses to examine separately how the benefits of early TXA treatment and the harms of late TXA treatment vary by SBP (≤75, 76–89, >89 mmHg); GCS score (severe 3–8, moderate 9–12, mild 13–15); and type of injury (penetrating versus blunt). Figure 2 shows the effects of early TXA treatment stratified by SBP, GCS and type of injury. There was no significant heterogeneity. Figure 3 shows the effects of late TXA treatment stratified by SBP, GCS and type of injury. Once again, there was no significant heterogeneity. As a more powerful sensitivity analysis, we tested for subgroup effects on the time by treatment interaction in a model including a three-way interaction term. There was no significant heterogeneity in the effect of TXA, according to SBP, GCS and type of injury, when time to treatment was taken into account.

## Discussion

Patients in the CRASH-2 trial who were treated with TXA within 3 h of injury were less likely to have blunt trauma and head injury and had lower SBPs than those treated after 3 h. However, these differences do not completely explain the strong time to treatment interaction, although the interaction was not as obvious in patients with a low GCS. When given within 3 h of injury, TXA reduces death due to bleeding regardless of injury type, GCS or blood pressure.

We have previously reported subgroup analyses of the effect of TXA according to SBP, GCS and type of injury [2]. All of these analyses were prespecified in the trial protocol. However, because they ignore the time to treatment interaction their value is limited. If TXA is either beneficial or harmful depending on how soon after injury the patient is treated, the overall effect regardless of time to treatment is neither biologically relevant nor generalisable since it depends largely on the proportion of patients treated early or late in the trial. Having identified that the effect of TXA depends on time to treatment, all other subgroup analyses should be conducted separately for early or late-treated patients as we have done here. The most striking observation from these analyses is the absence of heterogeneity. Early treatment appears effective and late treatment appears harmful in all subgroups. These analyses provide no support for the assertion that TXA is only indicated for patients with severe shock (SPB <75 mmHg) [4]. Early treatment is effective regardless of SBP.

Recent results suggest biological explanations for the time to treatment interaction. Early fibrinolysis is common after trauma and is associated with increased mortality [5, 6]. Tissue plasminogen activator (TPA), the enzyme that converts plasminogen to the fibrinolytic enzyme plasmin, is stored within the endothelium in Weibel-Palade bodies. Trauma triggers the release of TPA resulting in plasmin activation, fibrinolysis and profuse bleeding [7, 8]. TPA levels peak 30 min after injury and plasmin levels peak around 1 h [8]. By inhibiting fibrinolysis, TXA prevents early exsanguination [9]. However, the effects are short lived. Around 2 h after injury plasminogen activator inhibitor-1 (PAI-1) levels start to increase reaching a peak around 3 h [8]. PAI-1 inhibits fibrinolysis resulting in ‘fibrinolytic shutdown’ [10]. This might explain why the benefits of TXA treatment are limited to the first 3 h. The adverse effects of late TXA administration may be due to PAI-1-induced suppression of fibrinolysis and the onset of thrombotic disseminated intravascular coagulation (DIC). By inhibiting fibrinolysis TXA could worsen DIC. Although the pathology is thrombotic, due to the consumption of clotting factors, thrombotic DIC usually manifests clinically as bleeding [9].

The differential effects of TXA on TPA and urokinase plasminogen activator (UPA)-mediated plasminogen activation have also been proposed as a potential biological explanation of the ‘TXA paradox’ of time-dependent effects [11]. Although TXA inhibits TPA-mediated fibrinolysis, it stimulates fibrinolysis mediated by UPA [12]. The binding of TXA to plasminogen results in a conformational change in the plasminogen molecule that makes it more readily cleaved by UPA. The potential clinical importance of this finding is highlighted in animal models of the effect of TXA in closed head injury [13]. Two hours after injury, TXA treatment reduced intracranial bleeding consistent with the inhibition of TPA-mediated fibrinolysis [13]. However, when given 8 h after injury, when UPA levels were highest, TXA increased intracranial bleeding [13].

## Conclusions

All trauma patients with potentially life-threatening bleeding within 3 h of injury should be treated with TXA regardless of blood pressure, GCS or type of injury. Treatment after 3 h appears to increase the risk of death due to bleeding and, therefore, is contraindicated. Although it has been suggested that thromboelastography can be used to further subdivide patients into those that will benefit from TXA and those that will not, further research is needed [10, 14]. Whether TXA reduces mortality in isolated traumatic intracranial, gastrointestinal and postpartum bleeding and whether any such effect depends on time to treatment is open to question but will be informed by the results of large-scale clinical trials in these areas [15–17].

## Key messages

- Tranexamic acid reduces mortality in bleeding trauma patients but the effect appears to depend on time to treatment. Early treatment reduced bleeding deaths whereas late treatment increased the risk. We examined how patient characteristics vary by time to treatment and whether such variations explain the time-dependent treatment effect
- Patients treated within 3 h were less likely to have blunt trauma and head injury and had lower SBPs than those treated after 3 h. These differences do not completely explain the strong time to treatment interaction, although the interaction was not as obvious in patients with a low score on the GCS
- When given within 3 h of injury, TXA reduces death due to bleeding regardless of the type of injury, GCS or SBP. Treatment after 3 h increases the risk of death due to bleeding

## Additional files

Additional file 1: Interaction with time since injury stratified by type of injury. The graph shows how the treatment effect varies with time to treatment stratified by type of injury (blunt or penetrating). (PDF 6 kb)
Additional file 2: Interaction with time since injury stratified by Glasgow Coma Scale (GCS). The graph shows how the treatment effect varies with time to treatment stratified by GCS. (PDF 6 kb)
Additional file 3: Interaction with time since injury stratified by systolic blood pressure (SBP). The graph shows how the treatment effect varies with time to treatment stratified by SBP. (PDF 6 kb)
Additional file 4: Additional statistical analyses. These additional analyses were conducted in response to reviewer’s comments and use statistical models to examine potential confounding and interaction. (DOCX 15 kb)
Additional file 5: Consort trial profile. (DOCX 16 kb)
Additional file 6: List of ethics committees that approved the trial. List of ethics committees. (DOCX 21 kb)

## Abbreviations

CRASH-2 trial: Clinical Randomisation of Antifibrinolytic in Significant Haemorrhage trial
DIC: Disseminated intravascular coagulation
GCS: Glasgow Coma Scale
OR: Odds ratio
PAI-1: Plasminogen activator inhibitor-1
RR: Relative risk
SBP: Systolic blood pressure
TPA: Tissue plasminogen activator
TXA: Tranexamic acid
UPA: Urokinase plasminogen activator
