# Supplementary figures and images for: Tranexamic acid in bleeding trauma patients: an exploration of benefits and harms
Source: Trials. 2017 Jan 31;18:48. doi: 10.1186/s13063-016-1750-1 (PMC5282847; doi:10.1186/s13063-016-1750-1)

## Interaction with time since injury and Blunt Injury

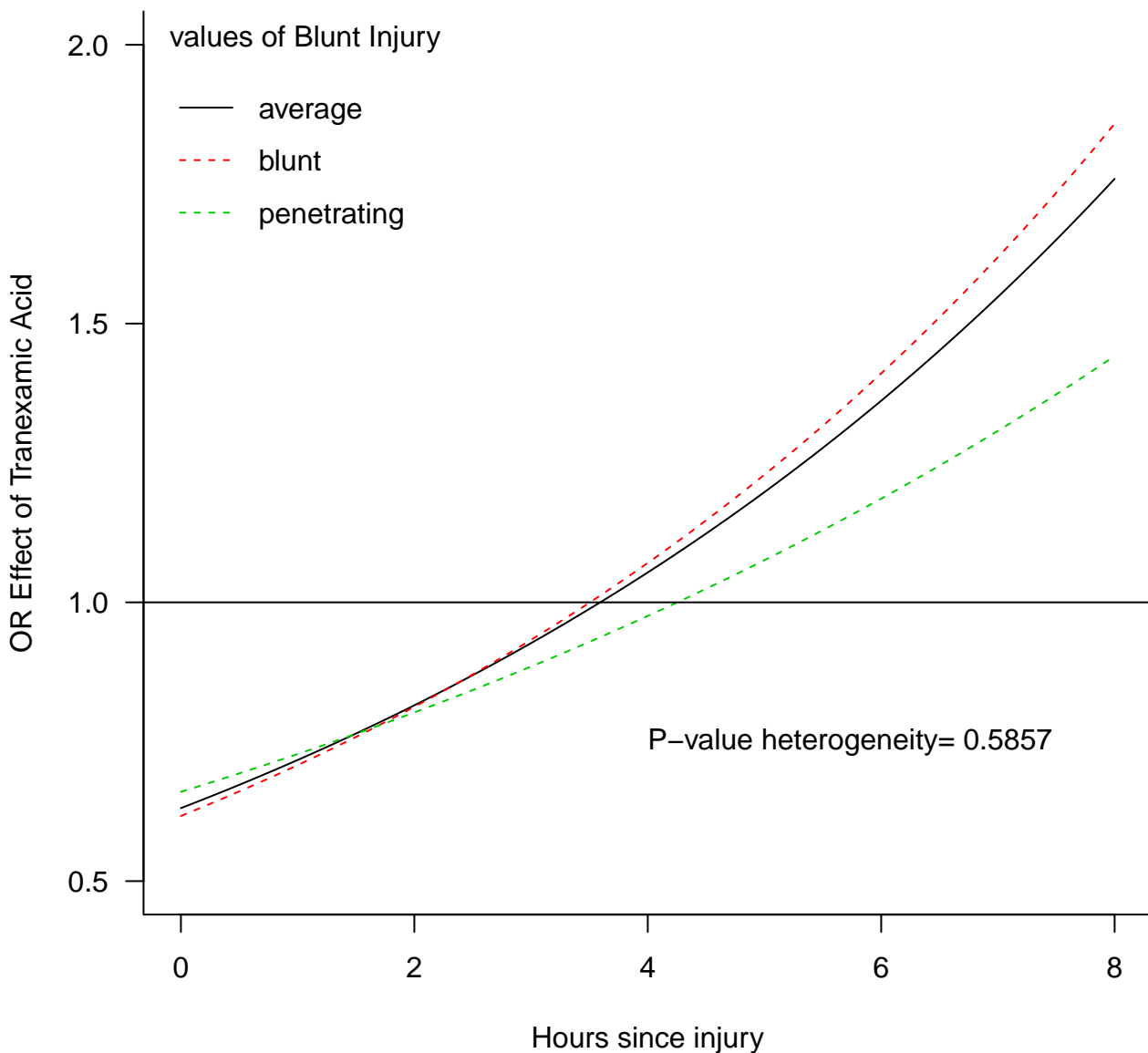

Supplement: Additional file 1: — Interaction with time since injury stratified by type of injury. The graph shows how the treatment effect varies with time to treatment stratified by type of injury (blunt or penetrating). (PDF 6 kb) [file 13063_2016_1750_MOESM1_ESM.pdf]

## Interaction with time since injury and G.C.S

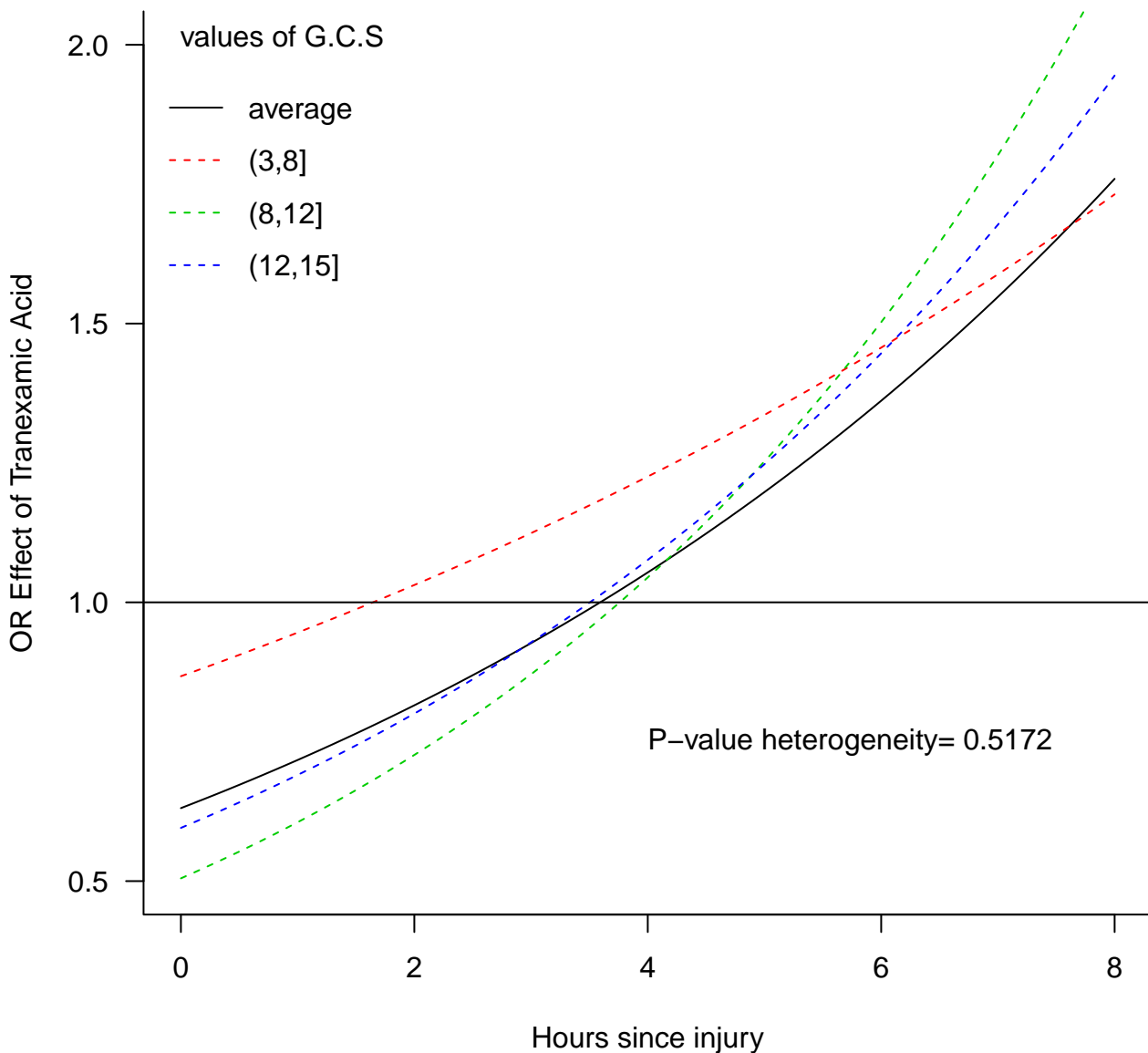

Supplement: Additional file 2: — Interaction with time since injury stratified by Glasgow Coma Scale (GCS). The graph shows how the treatment effect varies with time to treatment stratified by GCS. (PDF 6 kb) [file 13063_2016_1750_MOESM2_ESM.pdf]

## Interaction with time since injury and SBP (mmHg)

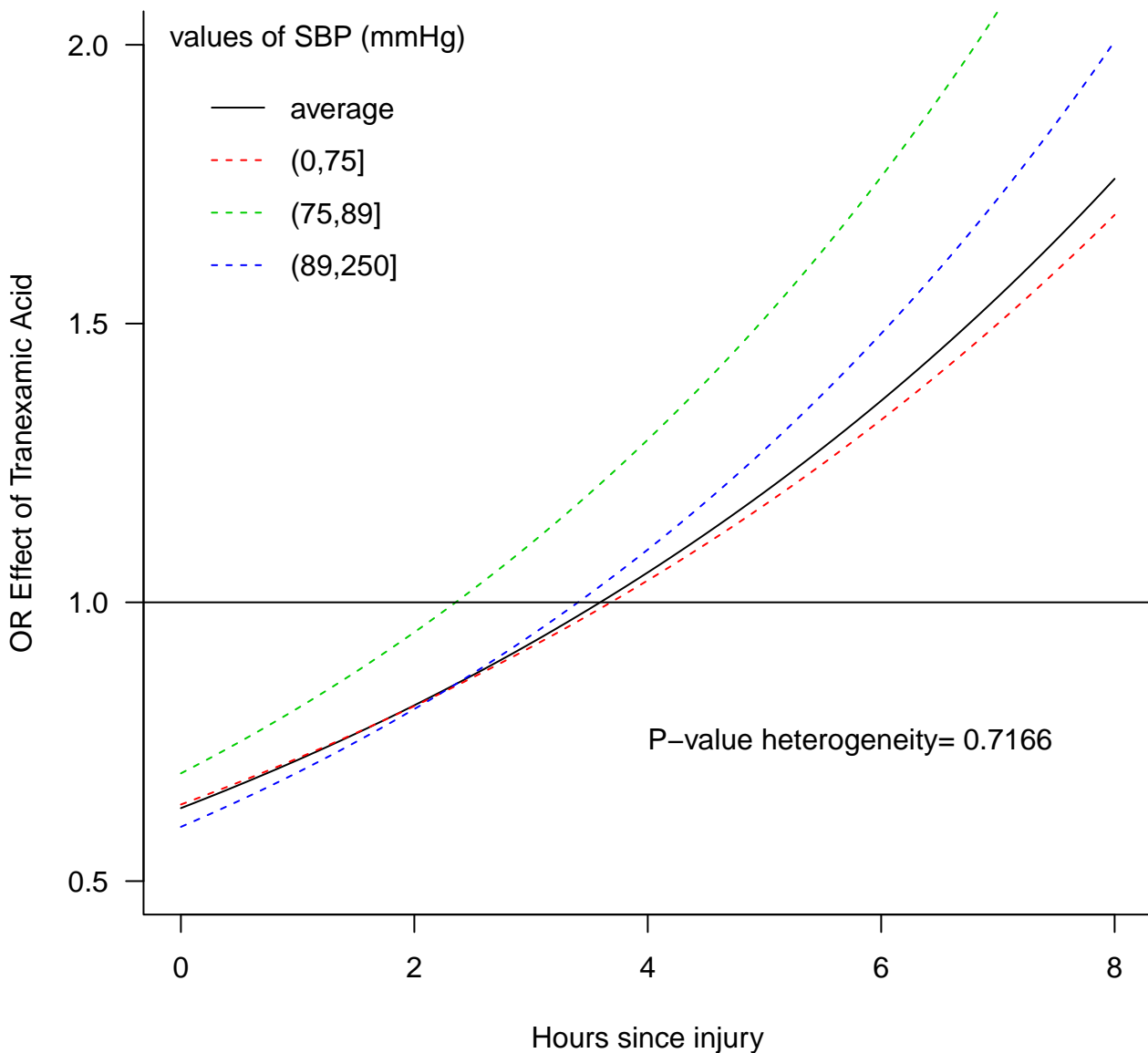

Supplement: Additional file 3: — Interaction with time since injury stratified by systolic blood pressure (SBP). The graph shows how the treatment effect varies with time to treatment stratified by SBP. (PDF 6 kb) [file 13063_2016_1750_MOESM3_ESM.pdf]
