# Supplementary material for: Tranexamic acid in bleeding trauma patients: an exploration of benefits and harms
Source: Trials. 2017 Jan 31;18:48. doi: 10.1186/s13063-016-1750-1 (PMC5282847; doi:10.1186/s13063-016-1750-1)
Supplement: Additional file 5: — Consort trial profile. (DOCX 16 kb) [file 13063_2016_1750_MOESM5_ESM.docx]

|  | Randomised =20,211 |  |
| --- | --- | --- |
| Allocated TXA 10,096 | *Treatment allocation* | Allocated Placebo 10,115 |
|  |  |  |
| Consent withdrawn 3 | *Withdrawal after randomisation* | Consent withdrawn 1 |
|  |  |  |
| Baseline data 10,093  Received allocated loading dose 9,955  Received allocated maintenance dose 9,490 | *Allocation* | Baseline data 10,114  Received allocated loading dose 9,989  Received allocated maintenance dose 9,475 |
|  |  |  |
| No follow-up 33 | *Follow-up* | No follow-up 47 |
|  |  |  |
| Analysed 10,060 | *Analysis* | Analysed 10,067 |

Additional file: Consort trial profile.
