# Supplementary material for: Tranexamic acid in bleeding trauma patients: an exploration of benefits and harms
Source: Trials. 2017 Jan 31;18:48. doi: 10.1186/s13063-016-1750-1 (PMC5282847; doi:10.1186/s13063-016-1750-1)
Supplement: Additional file 4: — Additional statistical analyses. These additional analyses were conducted in response to reviewer’s comments and use statistical models to examine potential confounding and interaction. (DOCX 15 kb) [file 13063_2016_1750_MOESM4_ESM.docx]

**Statistical Appendix:**

As a sensitivity analysis we tested for confounding of the time to treatment interaction by an analysis of the adjusted 2-way interaction. The results are shown below. The subgroups are categories of (S) systolic blood pressure, (G) Glasgow Coma Score and (I) type of injury. The adjusted time to treatment-time interactions terms are shown in **bold**. After controlling for confounders, the treatment-time interaction (E*T) remained in the case of models with SBP and type of injury type, although the interaction was not as obvious in patients with a low GCS.

| **Model Term** | **Risk Ratio** | **95% Confidence Interval** | **P value** |
| --- | --- | --- | --- |
| **Systolic blood pressure** |  | | |
| Treatment Group (E) | 0.838 | (0.755,0.930 ) | 0.001 |
| Time category (T) | 0.873 | (0.776,0.981) | 0.023 |
| SBP category (S) | 0.524 | (0.495,0.555) | <0.001 |
| **E*T** | **1.153** | **(1.012, 1.313)** | **0.032** |
| E*S | 1.025 | 0.954, 1.101 | 0.503 |
| T*S | 1.154 | 1.073. 1.240 | <0.001 |
| **Glasgow Coma Score** |  | | |
| Treatment Group (E) | 0.899 | (0.824, 0.980) | 0.016 |
| Time category (T) | 0.705 | (0.643, 0.773) | <0.001 |
| GCS category (G) | 0.425 | (0.392, 0.459) | <0.001 |
| **E*T** | **1.108** | **(0.977, 1.257)** | **0.109** |
| E*G | 0.974 | (0.881, 1.077) | 0.606 |
| T*G | 1.053 | (0.522, 0.587) | <0.001 |
| **Type of Injury** |  | | |
| Treatment Group (E) | 0.954 | (0.795, 1.145) | 0.614 |
| Time category (T) | 0.935 | (0.784, 1.115) | 0.457 |
| Injury Category (I) | 0.975 | (0.902, 1.054) | 0.522 |
| **E*T** | **1.156** | **(1.012, 1.321)** | **0.033** |
| E*I | 0.930 | (0.842, 1.026) | 0.146 |
| T*I | 0.986 | (0.892, 1.089) | 0.777 |

As a further sensitivity analysis, we tested for heterogeneity of the treatment effect by assessing the significance of the 3-way interaction. The three way interaction terms are for treatment group (E), time (T) and subgroup (systolic blood pressure (S), Glasgow Coma Score (G) and type of injury (I)). There was no significant heterogeneity.

| 3-way interaction (combined model) |  | | |
| --- | --- | --- | --- |
| **E*T*S** | **1.038** | **(0.902,1.194)** | **0.602** |
| **E*T*G** | **1.119** | **(0.909, 1.376)** | **0.289** |
| **E*T*I** | **1.000** | **(0.842, 1.186)** | **0.995** |
